# Development of reverse-transcription loop-mediated isothermal amplification assay for rapid detection and differentiation of dengue virus serotypes 1–4

**DOI:** 10.1186/s12866-015-0595-1

**Published:** 2015-11-14

**Authors:** Sheng-feng Hu, Miao Li, Lan-lan Zhong, Shi-miao Lu, Ze-xia Liu, Jie-ying Pu, Jin-sheng Wen, Xi Huang

**Affiliations:** Program of Immunology, Institute of Human Virology, Affiliated Guangzhou Women and Children’s Medical Center, Zhongshan School of Medicine, Sun Yat-sen University, 74 Zhongshan 2nd Road, Guangzhou, 510080 China; Key Laboratory of Tropical Diseases Control (Sun Yat-sen University), Ministry of Education, Guangzhou, 510080 China; Department of microbiology and immunology, Wenzhou, Medical University, Wenzhou, China

**Keywords:** Dengue virus, Dengue serotypes 1–4, Diagnostic accuracy, Reverse transcriptase loop-mediated isothermal amplification, Serotype detection

## Abstract

**Background:**

Dengue virus (DENV), the most widely prevalent arbovirus, continues to be a threat to human health in the tropics and subtropics. Early and rapid detection of DENV infection during the acute phase of illness is crucial for proper clinical patient management and preventing the spread of infection. The aim of the current study was to develop a specific, sensitive, and robust reverse transcriptase loop-mediated isothermal amplification (RT-LAMP) assay for detection and differentiation of DENV1-4 serotypes.

**Results:**

The method detection primers, which were designed to target the different DENV serotypes, were identified by inspection of multiple sequence alignments of the non-structural protein (NS) 2A of DENV1, NS4B of DENV2, NS4A of DENV3 and the 3′ untranslated region of the NS protein of DENV4. No cross-reactions of the four serotypes were observed during the tests. The detection limits of the DENV1-4-specific RT-LAMP assays were approximately 10-copy templates per reaction. The RT-LAMP assays were ten-fold more sensitive than RT-PCR or real-time PCR. The diagnostic rate was 100 % for clinical strains of DENV, and 98.9 % of the DENV-infected patients whose samples were tested were detected by RT-LAMP. Importantly, no false-positives were detected with the new equipment and methodology that was used to avoid aerosol contamination of the samples.

**Conclusion:**

The RT-LAMP method used in our study is specific, sensitive, and suitable for further investigation as a useful alternative to the current methods used for clinical diagnosis of DENV1-4, especially in hospitals and laboratories that lack sophisticated diagnostic systems.

## Background

Dengue virus (DENV), a member of the *Flaviviridae* family, is the most prevalent arbovirus in more than 100 countries within tropical and subtropical regions of the world [1]. There are four distinct serotypes, described as DENV1, DENV2, DENV3, and DENV4 [2]. DENV infection causes dengue fever, the more dangerous dengue hemorrhagic fever and dengue shock syndrome, all of which are very contagious [3]. According to a World Health Organization report, there are 50 million DENV infections and 500,000 cases of dengue hemorrhagic fever annually, the latter requiring hospitalization [4]. In 2014, there was an outbreak of dengue fever in China and countries within Southeast Asia, with more than 50,000 DENV-infected people in southern China. DENV infections are not only a threat to human health, but also cause huge economic losses to society.

The clinical characteristics of primary DENV do not involve bleeding or shock, and induce a life-long protective immunity to the homologous serotype responsible for the infection [5]. Because of antibody-dependent enhancement [6], upon reinfection with a different DENV serotype, antibodies against the virus are able to form a type of immune complex that activates the complement system resulting in immunopathologies [7], such as the pathogenesis of dengue fever and dengue shock syndrome. Without an effective vaccine to prevent DENV infection, multiple and sequential infections with DENV1-4 are to be expected for people living in regions where dengue is hyperendemic [6, 8].Therefore, rapid detection and differentiation of DENV serotypes are crucial for effective clinical diagnosis as well as for epidemiological investigation of this pathogen.

Early and rapid detection of DENV infection during the acute phase of illness are crucial for proper patient management and preventing the spread of infection. For microbiological diagnosis of DENV, several techniques have been developed; these include virus isolation, immunoassays, and biochemical tests using nucleotide probes [9–11]. Virus isolation is the “gold standard” for DENV detection. However, it is laborious and time consuming when used for routine clinical examination of patients. The presence of cross-reactive antigens shared by flaviviruses makes specific diagnosis of DENV by immunoassay not possible in most cases. Furthermore, because the antibodies produced in response to the virus do not occur at an early stage of the infection, the immunoassay methods are not suitable for early diagnosis. Nucleic acid detection is deemed to be a timely and effective method for diagnosis of DENV infection. Reverse transcriptase (RT)-PCR and real-time PCR methods have both been used widely for laboratory diagnosis because of their high sensitivities and specificities [12, 13]. However, they require specialized equipment, which restricts the popularization of this approach [14–16]. Hence, there is great demand for a rapid, simple, convenient, and appropriate method for use in resource-poor health clinics.

Loop-mediated isothermal amplification (LAMP), developed by Notomi in 2000, can achieve fast amplification of nucleic acid using only a water bath or heating block [17]. This method is a promising tool to meet the increasing need for a fast and easily performable pathogen detection assay [18]. LAMP can be used to determine the sex of animals [19], and establish if a food has been genetically modified [20]. Reverse transcriptase LAMP (RT-LAMP), which is based on amplification of reverse-transcribed cDNA, has the same sensitivity of DNA amplification by the standard LAMP method [21]. This method has been adopted for detecting various viruses [22–25], especially for detecting viruses that cause similar symptoms in a host and share a high degree of nucleotide homology among them [15, 26, 27]. Disappointingly, because of its high sensitivity, RT-LAMP is easily affected by aerosol pollution thereby resulting in false-positive samples. The need to prevent aerosol generation is of paramount importance when using RT-LAMP.

Early reports of DENV1-4 detection by RT-LAMP involved small numbers (<100) of clinical samples, use of the C-prM gene [28], serotype-specific regions of the 3′ untranslated region of the same gene [21, 29, 30], or the non-structural protein 1 (NS1) [31]. However, use of the same gene is not the best choice for all DENV1-4 genotypes because there may be high sequence identity among genotypes with the same serotype but low sequence variability among the four serotypes; this situation is relevant for the C-prM gene, its 3′-UTR [32], and NS1 [31]. The detection limits for DENV1-4 differ and the diagnostic accuracy was < 90 % because the DENV1-4 primers were limited to the same gene in earlier reports. Moreover, the aerosol pollution problem had not been solved in earlier reports. These deficiencies have hindered promotion of RT-LAMP for the detection of DENV.

In this study, a DENV1-4-specific RT-LAMP assay was developed and evaluated using serum from patients with DENV infections. The results showed that the RT-LAMP assay was specific, sensitive, and accurate. With new equipment and a method that avoids aerosol pollution, RT-LAMP has the potential to become a useful and reliable method for clinical diagnostics of DENV, especially in hospitals and laboratories that lack sophisticated diagnostic systems.

## Results

### Primer design and specificity assessment of RT-LAMP for DENV1-4

**Fig. 1:**
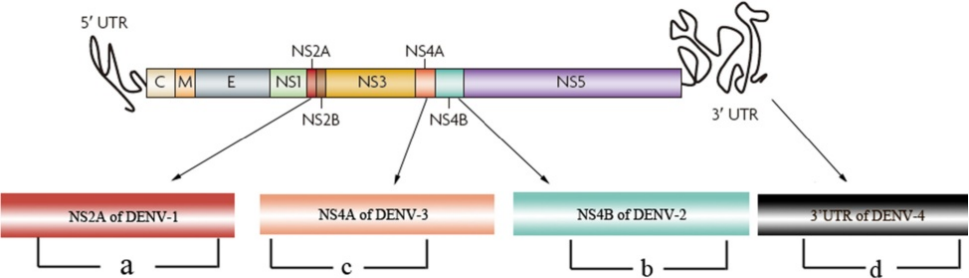
The DENV genome and the regions of the RT-LAMP primers of DENV1-4, respectively. **a**, the region of the RT-LAMP primer for DENV1; **b**, the region of the RT-LAMP primer for DENV2; **c**, the region of the RT-LAMP primer for DENV3; **d**, the region of the RT-LAMP primer for DENV4

The success of the RT-LAMP assay relies on the specificities of the primer sets that are used. A set of primers based on the optimal regions revealed by multiple sequence alignments (Fig. 1) was designed and used to evaluate the specificity of the DENV RT-LAMP assay; the primers are listed in Table 1.

**Table 1:** The primer sequences of RT-LAMP for DENV1-4

Primers	Sequence(5‘-3’)
DEN1-F3	TGTGTTCCTCCTTCTCATAATG
DENV1-B3	CAGACTCAATCCAATCGTAAGA
DENV1-FIP	CATCCTGTCTGAAGCATTGGCTGGACAATTGACATGGAATGATC
DENV1-BIP	CCTAGCTCTGATGGCCACTTTCTTCTCTAGATGTTAGTCTGCG
DENV1-LoopF	CCAACCATGATGCATAACCTG
DENV1-LoopB	ATGAGACCAATGTTCGCTGT
DENV2-F3	CACACTGGATAGCAGCTTC
DENV2-B3	CTATGTCCAGGATGTTGCTC
DENV2-FIP	GGCCACCACTGTGAGGATGAGAACACCCCAAGATAACC
DENV2-BIP	CAAACGAGATGGGTTTCCTGGATTGCTGGGTTGTAATGCTT
DENV2-LoopF	GGCTATGACAACGTAGGTCAA
DENV2-LoopB	ACGAAGAAAGATCTCGGATTGG
DENV3-F3	CCCGTCCAAGGACGTTAA
DENV3-B3	CTGCTGCGTTGTGTCATG
DENV3-FIP	ACGACGGAGCTACAGGCAGAAGAAGTCAGGCCCAAA
DENV3-BIP	GGGACGTAAAGCCTGGGAGCCTCTAACCACTAGTCTGCTA
DENV3-LoopF	GTTTGCTCAAACCGTGGC
DENV3-LoopB	AACCGTGGAAGCTGTACG
DENV4-F3	GCTCCTTTCGAGAGTGAAG
DENV4-B3	AGTACAGCTTCCTCCTGG
DENV4-FIP	CGTTATTGGCGGAGCTACAGGGAGGCTATTGAAGTCAGGC
DENV4-BIP	GGAGGCGTTAAATTCCCAGGGGTCTCCTCTAACCGCTAGT
DENV4-LoopF	CAGCACGGTTTGCTCAAG
DENV4-LoopB	CTGTACGCGTGGCATATTG

**Fig. 2:**
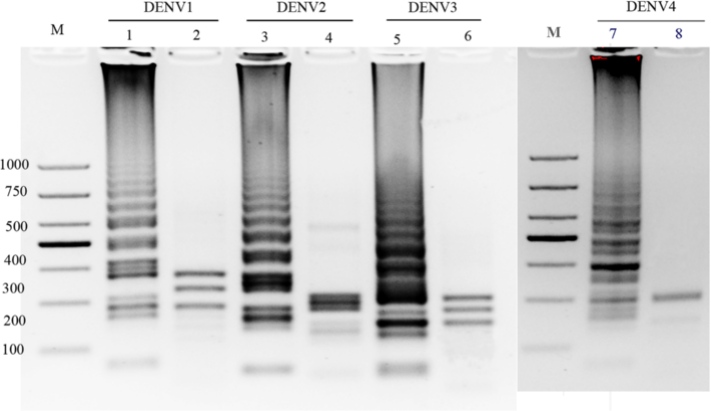
Agarose gel electrophoresis and restriction analysis of DENV serotype-specific RT-LAMP assay products on a 2 % agarose gel. M, DL1000 DNA ladder (TAKARA, Japan); 1, DENV1 RT-LAMP amplification; 2, *Xba*I restriction enzyme digestion of DENV1 RT-LAMP product, 190,235,280 bp respectively; 3, DENV2 RT-LAMP amplification; 4, *Spe*I restriction enzyme digestion of DENV2 RT-LAMP product, 190,205,220 bp respectively; 5, DENV3 RT-LAMP assay amplification; 6, *Bgl*II restriction enzyme digestion of DENV3 RT-LAMP product, 145,185,210 bp respectively; 7, DENV4 RT-LAMP assay amplification; 8, *Aba*I restriction enzyme digestion of DENV4 RT-LAMP product, 180,210 bp respectively

DENV1-4 was amplified successfully by the RT-LAMP method and its amplicons were observed as ladder-like patterns on agarose gels. The sizes of the resultant digested products were in agreement with the sizes predicted for DENV1-4 (Fig. 2). DNA sequencing of the digested products confirmed the specificity of the amplification (data not shown).

**Fig. 3:**
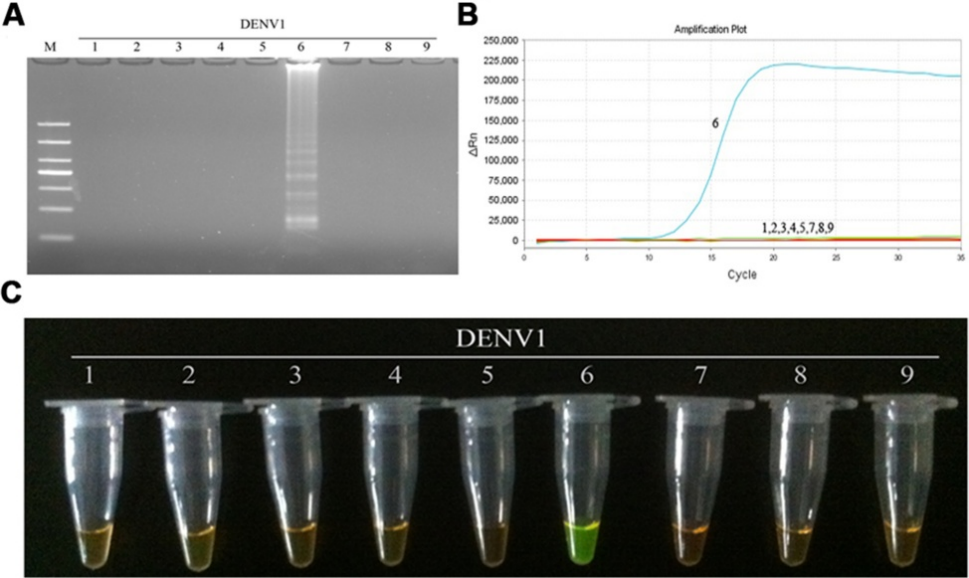
Specificity of RT-LAMP assay for the detection of DENV1. **a** Agarose gel electrophoresis analysis of the DENV1 RT-LAMP amplification product, showing the specificity of the primers. **b** The real-time monitoring over time for the DENV1 RT-LAMP reaction. **c** Visual inspection of the RT-LAMP specificity assay with SYBR Green I corresponding to the agarose gel electrophoresis analysis. 1, negative [43]; 2–3, DNA of HSV and EBV, respectively; 4–9, RNA of JEV, YFV and DENV1-4, respectively; M, DL1000 DNA Marker

**Fig. 4:**
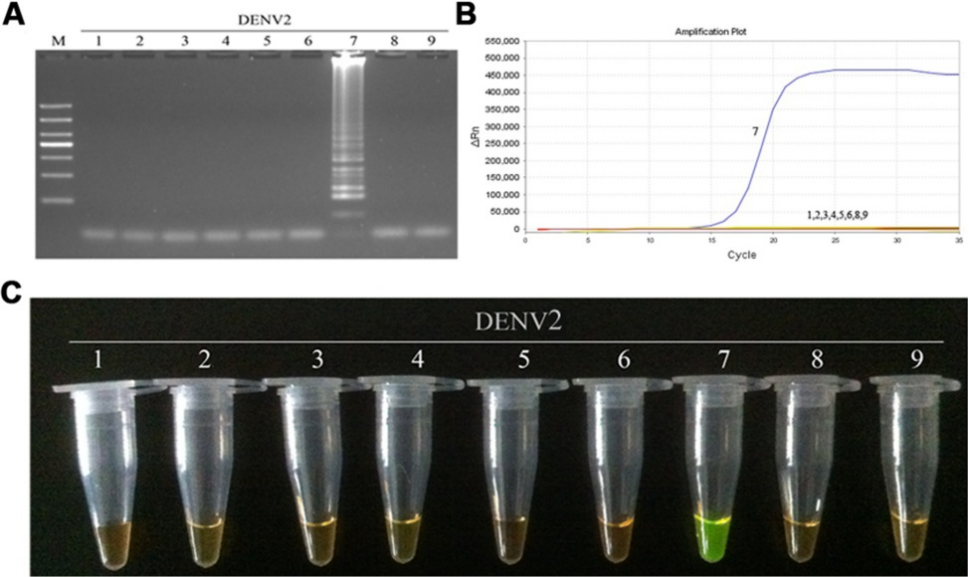
Specificity of RT-LAMP assay for the detection of DENV2. **a** Agarose gel electrophoresis analysis of the DENV2 RT-LAMP amplification product, showing the specificity of the primers. **b** The real-time monitoring over time for the DENV2 RT-LAMP reaction. **c** Visual inspection of the RT-LAMP specificity assay with SYBR Green I corresponding to the agarose gel electrophoresis analysis. 1, negative (water); 2–3, DNA of HSV and EBV, respectively; 4–9, RNA of JEV, YFV and DENV1-4, respectively; M, DL1000 DNA Marker

**Fig. 5:**
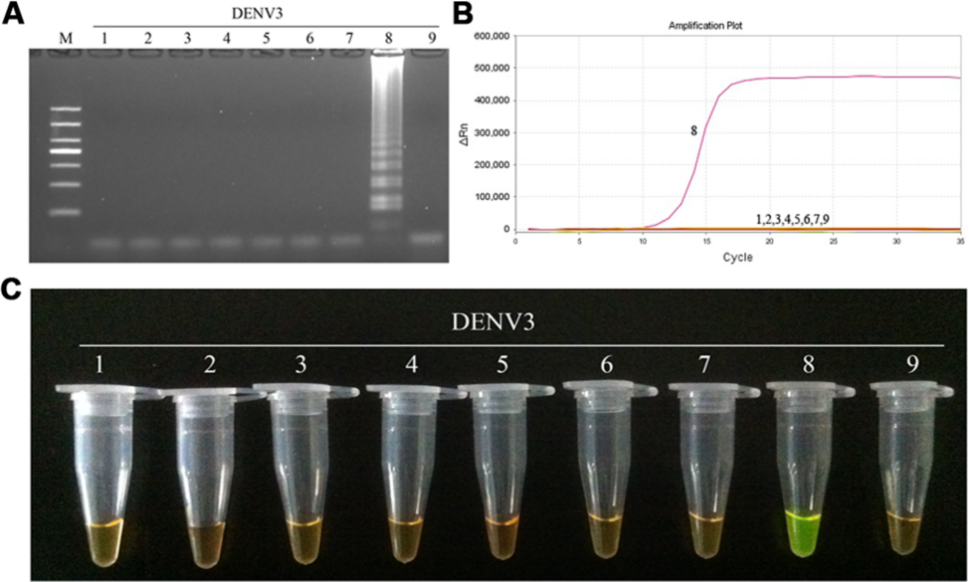
Specificity of RT-LAMP assay for the detection of DENV3. **a** Agarose gel electrophoresis analysis of the DENV3 RT-LAMP amplification product, showing the specificity of the primers. **b** The real-time monitoring over time for the DENV3 RT-LAMP reaction. **c** Visual inspection of the RT-LAMP specificity assay with SYBR Green I corresponding to the agarose gel electrophoresis analysis. 1, negative [43]; 2–3, DNA of HSV and EBV, respectively; 4–9, RNA of JEV, YFV and DENV1-4, respectively; M, DL1000 DNA Marker

**Fig. 6:**
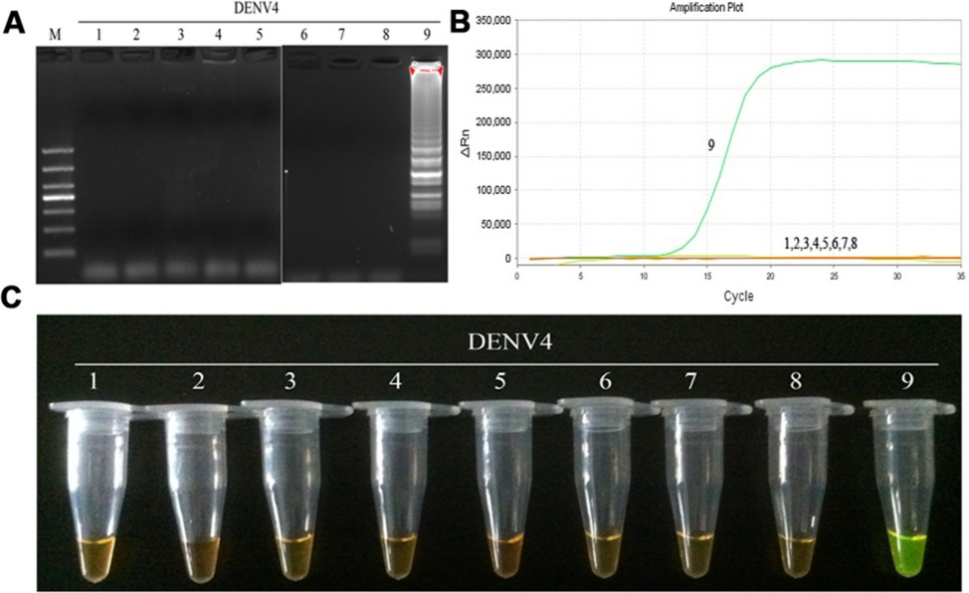
Specificity of RT-LAMP assay for the detection of DENV4. **a** Agarose gel electrophoresis analysis of the DENV4 RT-LAMP amplification product, showing the specificity of the primers. **b** The real-time monitoring over time for the DENV4 RT-LAMP reaction. **c** Visual inspection of the RT-LAMP specificity assay with SYBR Green I corresponding to the agarose gel electrophoresis analysis. 1, negative (water); 2–3, DNA of HSV and EBV, respectively; 4–9, RNA of JEV, YFV and DENV1-4, respectively; M, DL1000 DNA Marker

**Fig. 7:**
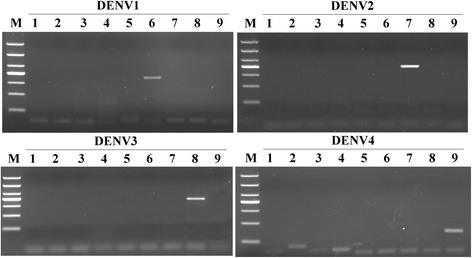
Specificity of RT-PCR assays for the detection of DENV1-4. Agarose gel electrophoresis analysis of the DENV1-4 RT-PCR amplification product, showing the specificity of the primers, DENV1-4-F3 and B3. (A-D) DENV1-4. 1, negative (water); 2–3, DNA of HSV and EBV, respectively; 4–9, RNA of JEV, YFV and DENV1-4, respectively; M, DL1000 DNA Marker

With the exception of the DENV1-4-positive RNA samples, Japanese encephalitis virus, yellow fever virus, herpes simplex virus, and Epstein-Barr virus were all used as negative controls. Each of the samples used in this study were tested ten times and the results were recorded. The DENV1-4 RT-LAMP primers showed high specificity by only amplifying their respective targets (Figs. 3, 4, 5 and 6). No cross-reactions and false-positive or false-negative results were obtained. Similarly, the F3 and B3 primers used by RT-PCR shown high specificity (Fig. 7).

### Sensitivity assessment of RT-LAMP for DENV1-4

**Fig. 8:**
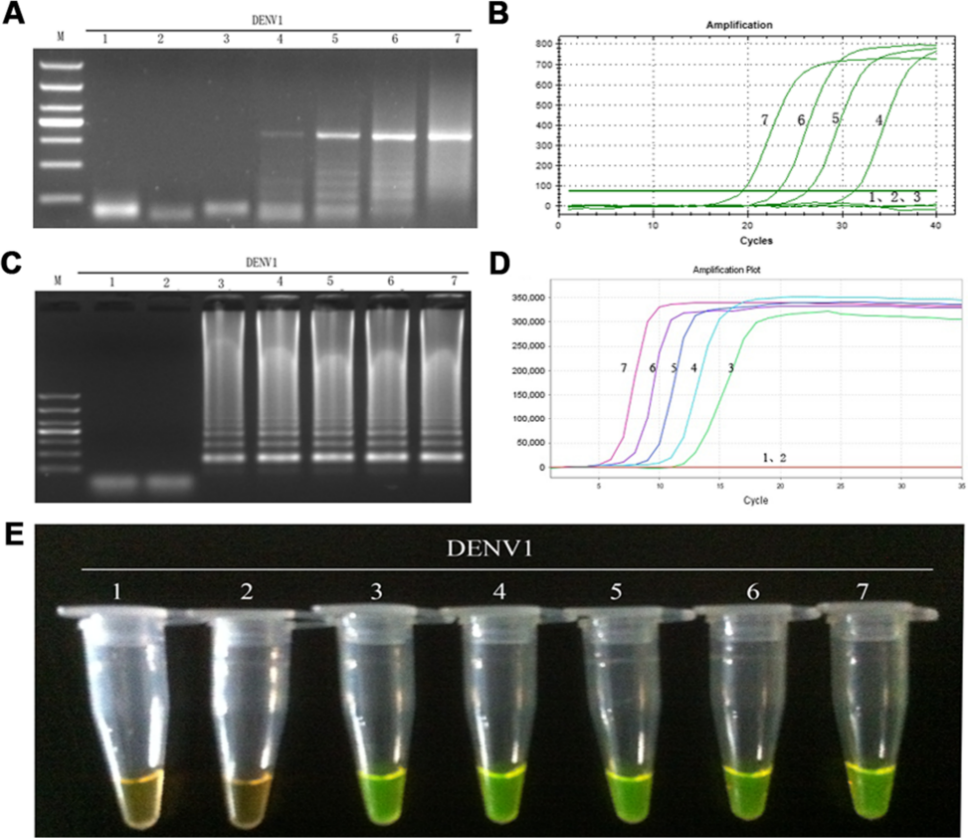
Comparison of the sensitivity of RT-PCR, real-time PCR and RT-LAMP for detection of DENV1. **a** Agarose gel electrophoresis analysis of detection limit of the RT-PCR assay for the detection of DENV1 RNA. **b** The real-time monitoring over time for detection limit of the real-time PCR assay for the detection of the DENV1 cDNAs. **c** Agarose gel electrophoresis analysis of the detection limit of the RT-LAMP assay for the detection of DENV1 RNA **d** Visual inspection of the RT-LAMP assay corresponding to agarose gel electrophoresis analysis. **e** The real-time monitoring of detection limit of the RT-LAMP assay. 1, negative [43]; 2–7, correspond to serial ten-fold dilutions of DENV1 RNA templates from 1 to 10^5^copies; M, DL1000 DNA Marker

**Fig. 9:**
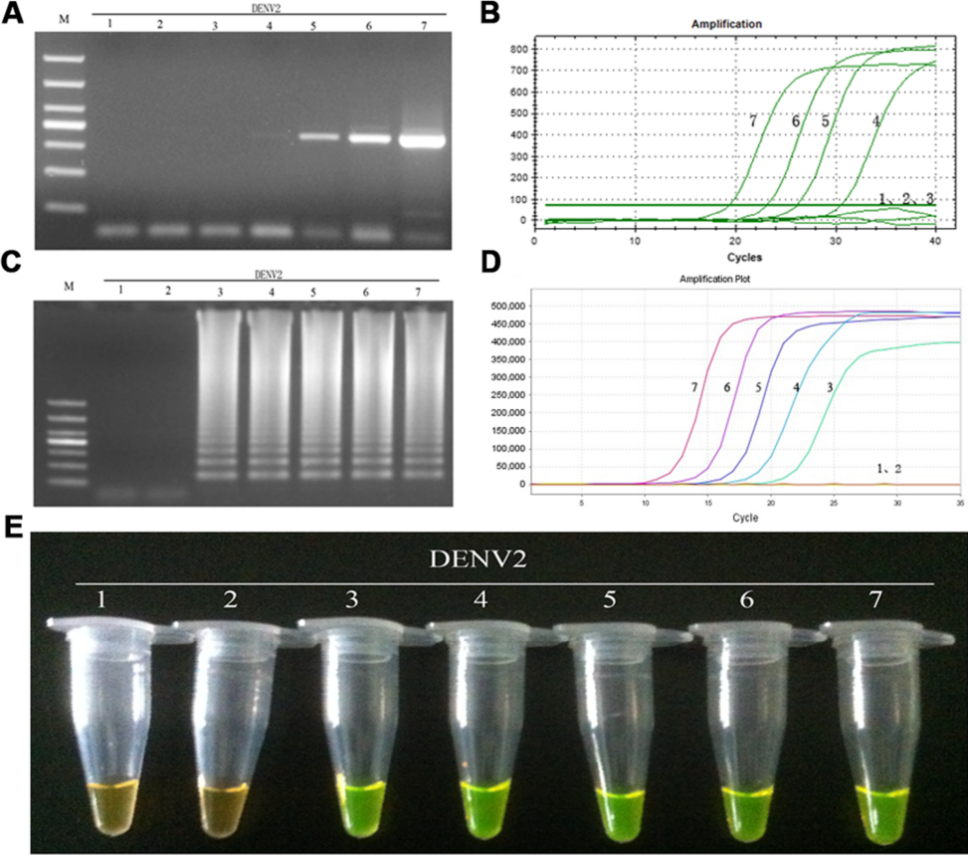
Comparison of the sensitivity of RT-PCR, real-time PCR and RT-LAMP for detection of DENV2. **a** Agarose gel electrophoresis analysis of detection limit of the RT-PCR assay for the detection of DENV2 RNA. **b** The real-time monitoring over time for detection limit of the real-time PCR assay for the detection of the DENV2 cDNAs. **c** Agarose gel electrophoresis analysis of the detection limit of the RT-LAMP assay for the detection of DENV2 RNA **d** Visual inspection of the RT-LAMP assay corresponding to agarose gel electrophoresis analysis. **e** The real-time monitoring of detection limit of the RT-LAMP assay. 1, negative (water); 2–7, correspond to serial ten-fold dilutions of DENV2 RNA templates from 1 to 10^5^copies; M, DL1000 DNA Marker

**Fig. 10:**
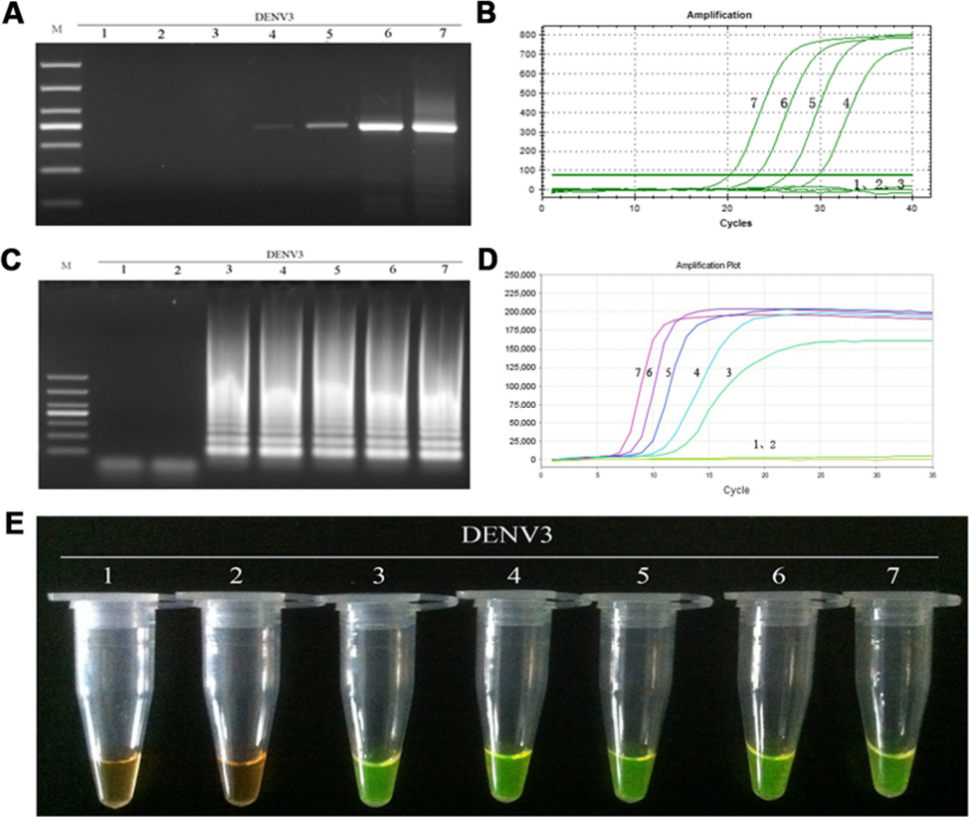
Comparison of the sensitivity of RT-PCR, real-time PCR and RT-LAMP for detection of DENV3. **a** Agarose gel electrophoresis analysis of detection limit of the RT-PCR assay for the detection of DENV3 RNA. **b** The real-time monitoring over time for detection limit of the real-time PCR assay for the detection of the DENV3 cDNAs. **c** Agarose gel electrophoresis analysis of the detection limit of the RT-LAMP assay for the detection of DENV3 RNA **d** Visual inspection of the RT-LAMP assay corresponding to agarose gel electrophoresis analysis. **e** The real-time monitoring of detection limit of the RT-LAMP assay. 1, negative [43]; 2–7, correspond to serial ten-fold dilutions of DENV3 RNA templates from 1 to 10^5^copies; M, DL1000 DNA Marker

**Fig. 11:**
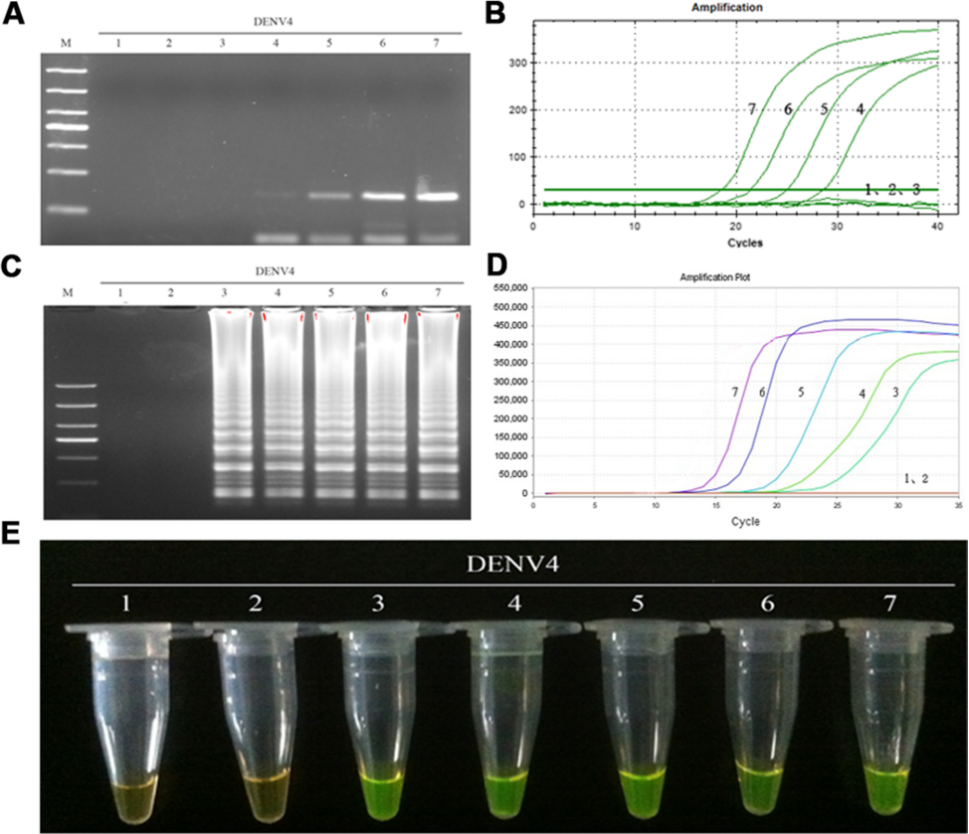
Comparison of the sensitivity of RT-PCR, real-time PCR and RT-LAMP for detection of DENV4. **a** Agarose gel electrophoresis analysis of detection limit of the RT-PCR assay for the detection of DENV4 RNA. **b** The real-time monitoring over time for detection limit of the real-time PCR assay for the detection of the DENV4 cDNAs. **c** Agarose gel electrophoresis analysis of the detection limit of the RT-LAMP assay for the detection of DENV4 RNA **d** Visual inspection of the RT-LAMP assay corresponding to agarose gel electrophoresis analysis. **e** The real-time monitoring of detection limit of the RT-LAMP assay. 1, negative (water); 2–7, correspond to serial ten-fold dilutions of DENV4 RNA templates from 1 to 10^5^copies; M, DL1000 DNA Marker

The sensitivity of the RT-LAMP method for the detection DENV1-4 was determined by testing 10-fold serially diluted viral genomic RNA templates with known numbers of nucleic acid copies, and comparing the assay with those of RT-PCR and real-time PCR. As a consequence, positive results for the RT-LAMP assay were detected at 10-copy templates of DENV1-4 (Figs. 8, 9, 10 and 11c-e), while the detection limits for RT-PCR and real-time PCR were about 100 copies (Figs. 8, 9, 10 and 11a and b). Thus, the detection sensitivity of the RT-LAMP assay for amplification of DENV1-4 was 10-fold more sensitive than those of RT-PCR and real-time PCR.

### Amplification efficiency of RT-LAMP for DENV1-4

**Fig. 12:**
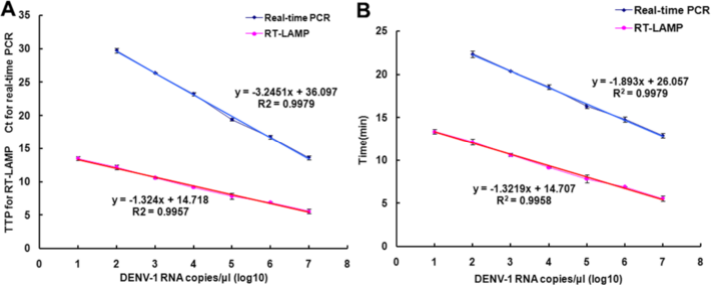
Comparison of the amplification efficiency and time of real-time PCR and RT-LAMP. **a** Standard curves generated by linear regression analysis of TTP of LAMP and Ct of real-time PCR measured for each amplification versus the log_10_ number of DENV-1 RNA or cDNA copies for each standard dilution. **b** Standard curves generated by linear regression analysis of the time for each amplification versus the log_10_ number of DENV-1 RNA or cDNA copies each standard dilution. Data are shown as mean ± standard error of the mean (S.E.M) at least three independent experiments. Ct, cycle threshold; TTP, time-to-positive

RT-LAMP products were detected by monitoring the increase in fluorescence by adding SYBR Green I to the RT-LAMP reaction mix. Quantitative analysis was obtained by measuring the time-to-positive (TTP) parameter [33], a biomarker similar to the cycle threshold of real-time PCR [34]. The standard curves were generated by linear regression analysis of TTP for RT-LAMP and the Ct of real-time PCR for each amplification reaction versus the log_10_ RNA copy number (Fig. 12a). Additionally, the time taken for fluorescence signal detection for each RT-LAMP and real-time PCR amplification was calculated according to TTP or Ct, respectively, after which the standard curves were generated by linear regression analysis (Fig. 12b). The time required by RT-LAMP was about half of that required by real-time PCR for every diluted concentration of DENV RNA, and the time required by the other serotypes was similar (date not shown). RT-LAMP was able to detect 10-copies of DENV RNA in 20 min, which should fit the requirement for rapid clinical diagnosis of DENV.

### Evaluation of RT-LAMP for clinical diagnosis of DENV1-4

The applicability of the RT-LAMP assay for detection and differentiation of DENV serotypes was validated by evaluating clinical strains of the virus and patient serum with DENV infection. Twenty clinical strains of DENV1, 30 clinical strains of DENV2, 15 clinical strains of DENV3 and 15 clinical strains of DENV4, were examined by the RT-LAMP assay, RT-PCR, and real-time PCR. As a result, the DENV1-4 detection rates had up to 100 % accuracy for RT-LAMP and real-time PCR, compared with 93 % by RT-PCR (Table 2). No cross-reactions between the serotypes were identified in this study.

**Table 2:** Comparative evaluation of DENV serotype-specific RT-LAMP assay with RT-PCR, and real-time PCR for detection of DENV stains and the patients’ serum samples

Type of case	Virus serotype	No. of samples Virus isolation	No. of samples positive by:		
			RT-LAMP	RT-PCR	Real-time PCR
Virus stains	DEN-1	20	20	18	20
	DEN-2	30	30	27	30
	DEN-3	15	15	15	15
	DEN-4	15	15	14	15
Total		80	80	74	80
Accuracy (%)			100	92.5	100
Patients’ serum samples	DEN-1	50	50	41	44
	DEN-2	60	59	52	54
	DEN-3	40	40	35	38
	DEN-4	40	39	33	36
Total		190	188	161	172
Accuracy (%)			98.95	84.74	90.53
Healthy		20	0	0	0
Total		20	0	0	0
False positive rate (%)			0	0	0

For the study on patient serum, 190 serum samples from patients confirmed to be infected by DENV by clinical diagnosis were treated to extract the total RNA. Additionally, 20 serum samples from healthy volunteers received the same treatment (negative controls). Among the positive samples, 98.9 % (188/190) were RT-LAMP assay positive, a value higher than 84.2 % (160/190) with RT-PCR and 90.5 % (172/190) with real-time PCR (Table 2), thus indicating that the sensitivity of the RT-AMP assay is higher than that of RT-PCR (*P* < 0.0001) and that of real-time PCR (*P* = 0.0003). None of the 20 healthy blood donors were positive for dengue viruses, as determined by the three methods. No cross-reactions or false-positive reactions were noted.

## Discussion

DENV is the most widely prevalent arbovirus in tropical and subtropical regions of the world [5]. There is an urgent need for fast and accurate clinical diagnosis and serotype differentiation of DENV1-4 to prevent and treat this viral infection. Conventional methods of DENV detection, which include virus isolation, immunoassay, RT-PCR, and real-time PCR [10], have many drawbacks in that they are time consuming to perform, require special equipment, and have a high propensity for cross-reaction of the DENV1-4 serotypes. In contrast, RT-LAMP can be performed using a water bath or a heating block under isothermal conditions [17], and is more sensitive and faster to perform than the other methods described herein. Some studies have reported the use of RT-LAMP for analyzing DENV. However, the detection limits for the DENV1-4 serotypes differed from each other and the diagnostic accuracy was < 90 % because the DENV1-4 primers were restricted to the same gene in the earlier reports [29–31, 35], although they could detect and differentiate DENV1-4 isotypes. In our study, we did not restrict the design of the primers to the same gene, but allowed different genes to be included in the search for optimal sequences with which to differentiate the different viral serotypes using multiple sequence alignments. This approach allowed us to establish an RT-LAMP method for detection of DENV 1–4 with higher sensitivity and greater diagnostic potential than RT-PCR and real-time PCR. The RT-LAMP reaction was sensitive enough to detect 10-copies of RNA template unlike RT-PCR and real-time PCR that each had detection limits of 100 copies. Scientific literature searches show that several studies have reported a higher sensitivity for real-time PCR than RT-PCR, but the detection limits of RT-PCR and real-time PCR can be discrepant in different detection systems, although both methods could detect 100 copies of DENV [36–39], which is consistent with our results.

In our diagnostic testing of the viral clinical strains and patients with DENV infections, the diagnostic rate RT-LAMP achieved was 100 % for the viral strains and 98.9 % for the patient samples, both values of which are better than that of RT-PCR (93 %, 84.2 %, respectively) and real-time PCR (100 %, 90.5 %, respectively). Encouragingly, the time required for confirmation of the results by the RT-LAMP assay was less than 25 min, just half of the time required by real-time PCR.

Because of its high sensitivity, RT-LAMP is more easily affected by aerosol pollution, a disadvantage ignored in previous studies. To avoid contamination, the tubes used for RT-LAMP reactions should not be opened; however, it is not feasible to add the SYBR Green I to the tube directly before the start of the LAMP reaction because a high concentration of SYBR Green I can inhibit this reaction [40]. Therefore, in our study, to avoid aerosol pollution, the SYBR Green I was dispensed directly onto the inner cover of the reaction tube. After the reaction was completed, the SYBR Green I was mixed with the RT-LAMP reaction mix by centrifugation. Furthermore, a new fluorescence-real time-monitoring instrument, ESE (DEAOU, Guangzhou, China) was used to eliminate aerosol pollution; this enabled the real-time monitoring of the RT-LAMP amplification to be realized using matched software. The risk of false-positives caused by aerosol contamination was minimized by using the improved methods and new equipment.

## Conclusions

The RT-LAMP method we established in this study is rapid, sensitive, and specific for the detection and differentiation of DENV1-4 serotypes. With new equipment and the application of our method to avoid aerosol contamination of the samples, the RT-LAMP method has potential for use in clinical laboratories where DENV assays are repeated many times. Moreover, it is convenient to quantitatively detect DENV1-4 by monitoring the fluorescence of the RT-LAMP reaction or by visually assessing the reaction using SYBR Green I for different requirements and test conditions.

## Methods

### Viruses

In our studies stains of viruses used were as follows, four standard dengue virus serotypes (DENV1, Hawaii; DENV2, New Guinea; DENV3, H87; and DENV4, H241), four DENV serotypes with clinical isolates and confirmation, JEV, YFV, HSV, EBV. DENV were propagated in *Aedes albopictus* clone C6/36 cells in our laboratory; JEV and YFV were contributed by Zhu Jiang hospital in Guangzhou in China; HSV and EBV were kindly provided by Professor Yan Yuan in our school. In addition, the DENV clinical stains were isolated and contributed by Zhu Jiang hospital and the CDC in Guangzhou, China.

### Dengue Virus Isolation and titers

Twenty to 200 μL of the initial serum sample of each patient were diluted with cell culture medium and inoculated onto confluent monolayers of C6/36 cells in 24 well plates. Serum samples were incubated for 4 h before being replaced by fresh medium and C6/36 cells were incubated at 35 °C. The clinical DENV strains were isolated after about 7 days.

The supernatants were collected and clarified by centrifugation (1000 g, 5 min). Viral concentrations were titered on C6/36 cells using the Reed-Muench method [41].

### Human patient serum samples and Ethics Statement

The serum samples were collected from patients who were febrile and suspected of having DENV 1–5 days after the presence of two or more of the symptoms viz. headache, eye pain, nausea, vomiting, rash, myalgia, abdominal pain. And then all the serum samples were screened by conventional diagnostic methods, the isolation of DENV, RT-PCR and IgM-capture ELISA. If there were two or more positive results, the patient would be confirmed with DENV infection. The number of DENV1-4 was counted in the process of the diagnosis.190 serum samples from patients with confirmed DENV infection were obtained in this study and frozen in −80 °C. Serum of 20 healthy blood donors were obtained from the physical examination center, the first affiliated hospital of Sun Yat-Sen University.

All serum samples used in this study were collected by Guangzhou CDC and appropriately anonymized. All individuals participating in the study gave written informed consent. Local ethical approval was obtained from Medical Ethics Committee of Guangzhou Center For Disease Control And Prevention (Guangzhou, China), and guidelines were followed for the use of clinical material and accession to diagnostic results.

### RNA extraction

Total RNAs were extracted from the supernatant of C6/36 cell infected DENV, and 200 μl patients’ sera using the QIAamp viral RNA mini kit (Qiagen, Hilden, Germany) according to the manufacturer’s instructions, respectively. The RNA was eluted from the QIA spin columns in a final volume of 50 μL of the elution buffer. Total RNAs were quantified using the Nano-Drop 2000c spectrophotometer (Thermo, Wilmington, DE). And then a little of every simple RNA was reversely transcribed in 25 μL reaction volume using random hexamer oligonucleotides with the reverse transcription system (Promega, Madison, WI). The rest of RNAs and the cDNA were stored at −80 °C until testing.

### Design of dengue virus serotype-specific RT-LAMP assay primers

The serotype-specific oligonucleotide primers used for RT-LAMP assay amplification of dengue viruses were designed from different regions (Fig. 1). The nucleotide sequences of the prototype strains of each dengue virus serotype were retrieved from GenBank (DEN-1, accession no. EU848545.1; DEN-2, accession no. AF 038403.1; DEN-3, accession no. M93130.1; and DEN-4, accession no AY947539.1), were aligned with the available sequences of other strains of each serotype to identify the potential regions which were the high sequence variabilities among DENV1-4 by using DNASIS software (Hitachi, Japan). And then the nucleotide sequences of the prototype strains of each DENV were aligned with the nucleotide sequences of the various clinical strains of each DENV to identify the optimal regions, which were the high sequence identities, in those potential regions. RT-LAMP assay primers were designed from the optimal region of each serotype using the software LAMP designer (Primer biosoft, America). A set of three pairs of primers comprising a pair of outer (F3 and B3), a pair of inner (FIP and BIP), and a pair of loop primers (FLP and BLP) that recognize eight distinct regions on the target sequence was designed. All the primers (Table 1) were selected based on the criteria described by Notomi et al. and then assessed for specificity before use in RT-LAMP assays with a BLAST search in the GenBank.

### RT-LAMP assays

The RT-LAMP reaction was carried out in a total 25 μL reaction mixture containing 40 pM each of primers FIP and BIP, 5 pmol each of outer primers F3 and B3, 20 pM each of FLP and BLP, 1.4 mM deoxynucleoside triphosphates, 0.8 M betaine, 0.1 % Tween 20, 10 mM (NH_4_)_2_SO_4_, 8 mM MgSO_4_, 10 mM KCl, 20 mM Tris–HCl, pH 8.8, 16 U of *Bst* DNA polymerase (New England Biolabs), 0.125 U of avian myeloblastosis virus reverse transcriptase (Invitrogen), and different copies of genomic RNA templates in sensitivity assays or about 100 ng of target RNA in in the others experiments, which were incubated at 63 °C for 45 min.

### Detection of RT-LAMP products

For naked-eye detection, 1.0 μL of 10^−1^ diluted SYBR Green I (Takara Bio Inc., Otsu, Japan) was dropped on the inner cover of the reaction tube to avoid the aerosol pollution, and then mixed the SYBR Green I and the reaction mixture to observe the color. For the electrophoretic analysis, 2 μL of reaction mixture was loaded on 2 % agarose gel. The gel was stained with ethidium bromide and assessed photographically under UV light. To confirm a structure of the RT-LAMP product, the amplification products was digested with restriction enzyme and was analyzed by the electrophoresis. For quantitative detection, real-time monitoring of RT-LAMP was performed using ESE (DEAOU, Guangzhou, China), a kind of new fluorescence-real time-monitoring instrument, with 8 wells and the positive and negative of the amplification shown on the instrument screen. SYBR Green I was used as a source of fluorescence. The original solution of SYBR Green I was diluted into 25× and then added 1.0 μl in the 25 μl RT-LAMP reaction mixture. All amplifications and detections were carried out in ESE. Accumulation of RT-LAMP products was detected by monitoring the increase in fluorescence of dsDNA-binding SYBR Green at every 1 min for 35 min under isothermal condition at 63 °C. The datas of real time RT-LAMP were analyzed by using the ESE software (DEAOU, Guangzhou, China).

### Specificity of RT-LAMP assays

The RT-LAMP production was digested with *Xba*I for DENV-1, *Bgl* II for the DENV-2, *Spe*I for DENV-3, *Apa*I for DENV4. The amplification products and the corresponding digests were analyzed by electrophoresis on a 2 % agarose gel, stained with ethidium bromide. The authenticity of the amplified products was also verified by nucleotide sequencing of digested products. Cross-reactivity was evaluated within the four dengue virus serotypes, two other *Flaviviruses*, JEV and YFV as well as two DNA virus, HSV and EBV, having a far away genetic relationship with dengue virus.

### Viral genomic RNA quantification

In vitro-transcribed RNA was used as the copy number control to quantify RNA templates of DENV1-4 as previously described [42]. Briefly, templates were developed by amplifying DENV1-4 using F3 and B3 prime pairs in the DENV1-4 RT-LAMP assay and cloned into a TOPO TA vector. Target RNA was transcribed with T7 RNA polymerase using AmpliScribe T7 Flash Transcription Kit. The resulting RNA was quantified by spectrophotometry and expressed as copy per mL (copy/mL). And then, the quantified RNA templates were diluted down to 10^5^ copies/mL to be used in followed assays.

### Sensitivity of the RT-LAMP assays

The sensitivity of RT-LAMP assays was carried out through ten-fold serially diluted viral genomic RNA templates with the known nucleic acid copies.

### RT-PCR assays

Each of viral samples was amplified in a 25 μL reaction containing 5 μL 5 × reaction buffer, 1 μL 25 mM MgSO4, 0.5 μL 10 mM dNTP Mix, 0.25 μL each of 50 μM primer F3 and B3 mixture, 0.5 μL 5U/μL AMA, 0.5 μL 5 U/μL *Tfl* DNA polymerase, 1 μL different copies of genomic RNA templates, and RNase- free ddH_2_O. Amplification by RT-PCR was performed using a Professional Thermocycler (Biometra-Göttingen, Germany). The thermal profile consisted of a 45 min reverse transcription step at 48 °C followed by 2 min of Taq polymerase activation at 94 °C, and then 35 cycles of PCR (94 °C for 30 s, annealing temperature 55 °C for 60 s, and 68 °C for 2 min).

### Real-time PCR assays

Viral RNA was reversely transcribed into cDNA as described above. Real-time PCR was performed in a 25 μL reaction containing 1 μL cDNA templates, 10 μL 2× SYBR® Premix Ex Taq™(TaKaRa), 2 μL 10 μM F3, 2 μL 10 μM B3, and nuclease-free ddH2O. The CFX96 Real-Time PCR System (Bio-Rad, CA) was programmed to denature the samples for 5 min at 95 °C, followed by 40 cycles of 95 °C for 15 s, 59 °C for 30 s, and 72 °C for 30 s.

### Statistical analysis

All statistical analysis was performed using IBM SPSS Statistics, version 21 (IBM Corporation, New York, United States). Chi-square test (McNemar’s exact test, two-tailed) was performed to evaluate and compare the sensitivity of all molecular and serological methods used. In the present study, the *p*-value <0.001 was used to suggest significant results. The diagnostic performance of RT-LAMP assay as compared to RT-PCR and Real-time PCR was calculated using web based Medcalc easy-to-use statistical software (https://www.medcalc.org/calc/diagnostic_test.php).

## Abbreviations

Ct: cycle threshold
DENV: dengue virus
DHF: dengue hemorrhagic fever
DSS: dengue shock syndrome
EBV: Epstein-Barr virus
HSV: herpes simplex virus
JEV: Japanese encephalitis virus
NS DF: dengue fever
RT-LAMP: reverse transcription-loop-mediated isothermal amplification
TTP: time-to-positive
YFV: yellow fever virus
